# Exploring cerebral small vessel disease signatures in familial Parkinson’s disease

**DOI:** 10.1038/s41598-026-69989-z

**Published:** 2026-09-08

**Authors:** Bigyan Mahat, Bimala Malla, Marco Foddis, Dieter Beule, Jose Bras, Rita Guerreiro, Vasilis Kola, Hans-Michael Schmitt, Matthias Endres, Celeste Sassi

**Affiliations:** 1 Department of Experimental Neurology, Center for Stroke Research Berlin (CSB), Charité – University of Medicine, Corporate Member of Freie University Berlin, Humboldt-University Berlin, and Berlin Institute of Health, Charitéplatz 1, D-10117 Berlin, Germany; 2 Department of Neurology, Werner Forssmann Hospital, Eberswalde, Germany; 3 Core Unit Bioinformatics, Berlin Institute of Health, BIH, Berlin, Germany; 4 Division of Psychiatry and Behavioral Medicine, Michigan State University College of Human Medicine, Grand Rapids, MI USA; 5 Department of Neurology, Charité – University of Medicine Berlin, Corporate Member of Freie University Berlin, Humboldt-University Berlin, and Berlin Institute of Health, Berlin, Germany

**Keywords:** Parkinson’s disease (PD), Vascular parkinsonism (VP), Parkinson Mendelian genes, Cerebral small vessel disease (cSVD), White matter hyperintensities (WMH), Whole exome sequencing (WES)., Diseases, Genetics, Neurology, Neuroscience

## Abstract

Familial Parkinson’s disease (PD) and vascular parkinsonism (VP) present overlapping features and may co-exist. To investigate whether PD and VP may share a potential pathogenic link and to what extent white matter hyperintensities may influence PD phenotype, we used the modified Scheltens scale and presented a descriptive and exploratory analysis of the classic neuroradiological features of cerebral small vessel disease (cSVD) in the axial T2-FLAIR MRI sequences in a cohort of 104 familial PD and PD prodromal patients and 48 age-matched controls from the PPMI publicly available database. We next performed whole exome sequencing to examine the protein coding variability in the main PD-causing and risk genes (*VPS35*, *DJ1*, *PINK1*, *ATP13A2*, *PRKN*, *SNCA*, *LRRK2*, *GBA*, *MAPT*, *LAMP3*, *STK39*) in a cohort of 96 patients with familial cSVD and 243 elderly healthy individuals (HEX database). In this cohort, patients with familial and prodromal PD present a moderate burden of superficial frontal white matter hyperintensities (p-value = 3.46e-06, Bonferroni-corrected), linked to a mild reduction of motor and cognitive function and an increased *LRRK2* p.G2019S and p.R1441C variant penetrance, and bilateral basal ganglia periventricular enlarged spaces (p-value = 2.64e-03, Bonferroni-corrected). Moreover, one-third of familial PD patients displayed a burden of lacunar thalamic strokes (p-value = 0.058), associated with a moderate hypokinetic-rigid syndrome. Finally, we report no known pathogenic coding variant in the main PD causative genes and risk factors in a cohort of 96 early-onset cSVD Caucasian patients. Our study adds to the understanding of potential cSVD hallmarks within this familial *LRRK2*, *GBA* and *SNCA* PD-PD prodromal cohort.

**Supplementary Information:**

The online version contains supplementary material available at https://doi.org/10.1038/s41598-026-69989-z.

## Introduction

Familial Parkinson’s disease (PD) and vascular parkinsonism (VP) present overlapping clinical (rigidity and bradykinesia, cognitive impairment to severe dementia) and to a different extent neuroradiological (white matter hyperintensities) and neuropathological (depigmentation of the substantia nigra, cortical atrophy) features. Both conditions may co-exist particularly in elderly patients and are frequently misdiagnosed^1–2^ (Table S1).

Vascular parkinsonism is often caused by sporadic, severe, diffuse progressive cerebral small vessel disease (cSVD). Other less common causes of VP include Mendelian cSVDs, such as Cerebral Autosomal Dominant Arteriopathy with Subcortical Infarcts and Leukoencephalopathy (CADASIL), Cerebral Autosomal Recessive Arteriopathy with Subcortical Infarcts and Leukoencephalopathy (CARASIL), and retinal vasculopathy with cerebral leukodystrophy (RVCL). More rarely, Mendelian leukodystrophies such as hereditary diffuse leukoencephalopathy with spheroids (HDLS) leading to a reduction of thalamo-cortical drive may also cause VP^2,3^ (https://www.omim.org/) (Table S2).

However, despite the comprehensive body of both clinical and experimental evidence describing white matter macro- and microstructural changes in PD patients and correlating these to the progression of diverse clinical PD phenotypes^4–5^, *LRRK2* p.G2019S variant leading to abnormalities in arterial cerebral blood flow in mouse experimental models^6^, a potential common pathogenic ground between familial PD and cSVD has not been extensively and systematically investigated. Moreover, although brain vascular lesions have been described in almost 25% of patients with idiopathic PD^7^, their contribution to the clinical phenotypic spectrum remains to be unveiled.

**Fig. 1:**
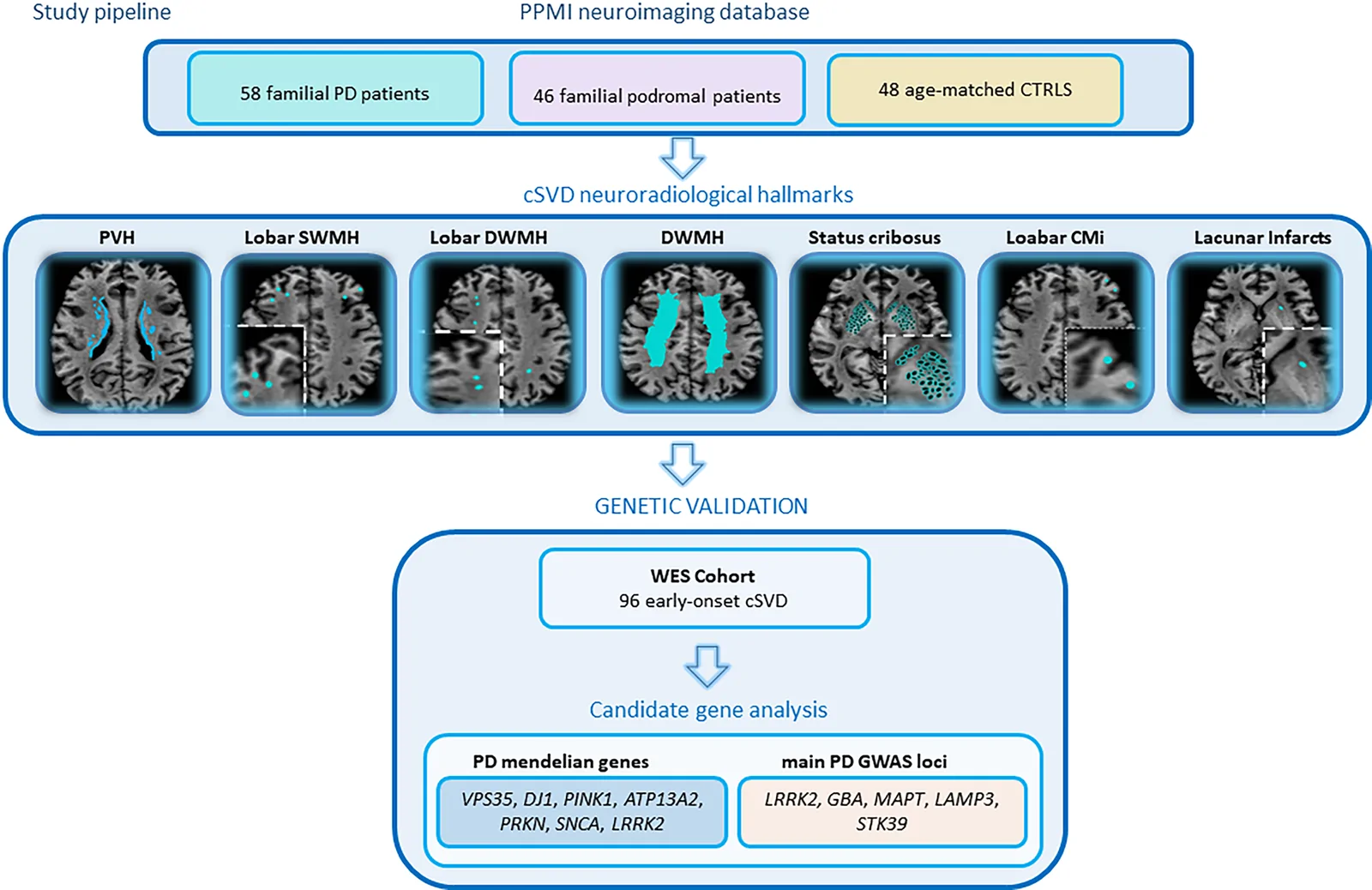
Study pipeline. T2-FLAIR MRI scans of a cohort of 152 individuals (58 familial PD patients, 46 familial prodromal PD patients and 48 age-matched controls) from the Parkinson’s Progression Markers Initiative (PPMI) database, were screened for the main cerebral small vessel disease neuroradiological hallmarks: peri-ventricular hyperintensities (PVH); lobar superficial white matter hyperintensities (SWMH); lobar deep white matter hyperintensities (DWMH); status cribrosus; lobar cortical small-and micro-infarcts (Si and Mi) and lacunar infarcts. To investigate the hypothesis that PD main genetic causative and risk factors may have played a critical role in cSVD, we performed whole exome sequencing (WES) on a cohort of 96 early-onset familial small vessel disease Caucasian patients from the US and in 243 elderly and neuropathologically proven controls from the publicly available HEX Database (https://www.alzforum.org/exomes/hex) and screened protein coding rare genetic variability in the main PD causative genes and genome wide association study (GWAS) loci.

Therefore, we used a modified Scheltens scale and screened the classical cSVD neuroradiological biomarkers (periventricular hyperintensities, lobar superficial white matter hyperintensities, lobar deep white matter hyperintensities, status cribrosus, lobar cortical small-microinfarcts, lacunar infarcts^8–9^, Table S3) in T2-FLAIR MRI sequences of 58 familial PD and 46 familial PD prodromal patients and 48 age-matched controls from the PPMI publicly available database^10^(Table 1, Table S4). Next, we performed exome sequencing on a cohort of 96 familial cSVD Caucasian patients from the US to investigate in this cohort protein coding variability in the most common PD Mendelian genes (*VPS35*, *DJ1*, *PINK1*, *ATP13A2*, *PRKN*, *SNCA*, *LRRK2*) and risk factors (*LRRK2*,* GBA*, *MAPT*, *LAMP3*, *STK39*) (https://www.omim.org/) (Fig. 1).

**Table 1:** PPMI MRI cohort used in this study

Pheno.									Cardiovascular risk factors (%)*							
	*N*. pat/ CTRLS	AAO (SD)	F (%)	*N*. fam. Cases (%)	LRRK2 (%)	GBA (%)	SNCA (%)	APOE ε2 (%)	Hypert. (%)	DMT 2 (%)	Hyperlipid (%)	AF (%)	CAD (%)	Valve prolapse/ regurgitation (%)	PFO (%)	Hypercoag.(%)
PD	58	60 (32–82)	22 (38)	58 (100%)	41/104 (39)	28/104 (27)	2/104 (2)		8/14 (57)	4/14 (28)	7/14 (50)	4/14 (28)	1/14 (7)	0/14 (0)	1/14 (7)	1/14 (7)
Prod. patients	46	63 (34–77)	27 (59)	46 (100%)				16/104 (15)	11/33 (33)	4/18 (22)	14/18 (77)	1/18 (5)	1/18 (5)	0/18 (0)	0/18 (0)	0/18 (0)
CTRLS	48	61 (32–81)	17 (35)	0				4/48 (8)	4/18 (22)	2/18 (11)	6/18 (33)	1/18 (5)	0/18 (0)	3/18 (16)	0/18 (0)	0/18 (0)

Pheno., phenotype; PD, Parkinson’s disease; Prod, prodromal; CTRLS, controls; N., number; pat, patients; AAO, age-at onset; SD, standard deviation; F, female; N, number; fam, familial; Hypert., hypertension; DMT 2, diabetes mellitus type 2; Hyperlipid, hyperlipidemia; AF, atrial fibrillation; CAD, coronary artery disease; PFO, pervium foramen ovale; Hypercoag., hypercoagulability; * Data available for 33/104 (32%) Parkinson’s disease and prodromal patients.

## Materials and methods

### MRI study cohort

Fifty-eight familial Parkinson’s disease patients (females = 22, mean age at onset 60 years [range: 32–82]), 46 familial prodromal PD patients (females = 27, mean age at of onset 63 years [range: 34–77]) and 48 age-matched healthy controls (females = 17, mean age at onset 61 years [range: 32–81]) were obtained from the Parkinson Progression Marker Initiative (PPMI) database (Table 1, Table S4). We downloaded data on PD patients from the PPMI database in December 2021.

The PPMI study is well described at www.ppmi-info.org^10^. Briefly, PPMI is an observational, comprehensive multi-center study designed to identify biomarkers of PD with the goal of improving evaluation of disease-modifying therapeutics^11^. Healthy control subjects included in PPMI were above 30 years of age, had never been diagnosed with any major neurological disorder and had no first-degree relatives with idiopathic PD. Familial PD and familial prodromal PD patients were defined as having at least one first-degree relative with idiopathic PD, as previously described ^10,12^.

The enrollment of PD and PD prodromal cases began in 2017. Among the inclusion criteria for the PPMI PD-PD prodromal cohort were protein-coding pathogenic variants in *LRRK2*, *SNCA* and *GBA* and the selection of causative genes was based on the main and well established PD genetic causative and risk factors until 2017 discovered: *LRRK2*, *SNCA* and *GBA*^13^.

To date, *LRRK2* mutations represent the most common genetic autosomal dominant cause of PD. *LRRK2* mutations display an incomplete and age-related penetrance and are found both in familial (5–6%) and sporadic (1%) PD cases. *GBA* coding mutations are detected in 5–30% of all PD patients and therefore are significant, common and age-dependent genetic risk factors for PD^14^. Finally, pathogenic *SNCA* mutations may be found both in autosomal dominant familial (less than 0.5%) and in sporadic PD cases (up to 0.01%)^14^.

Moreover, *SNCA*, *LRRK2*, and *GBA* have been shown to contain pleomorphic alleles with variable frequency and effect, from rare, highly penetrant variants to common risk alleles with small effect sizes, therefore being critical genes not only for the familial PD forms but providing also a critical insight into the sporadic form of the disease^15^.

Given the critical role of *LRRK2*, *GBA* and *SNCA* in PD pathogenesis, we focused on rare (Minor Allele Frequency [MAF] < 1%) known pathogenic coding variants in these genes, including both rare pathogenic mutations leading to late-onset PD (*LRRK2* and *GBA*, 64%) and early-onset PD (*SNCA*, 2%). Therefore, PD and PD-prodromal subjects included in the study presented mean age at onset of 60 years (range: 32–82) and 63 years (range: 34–77), respectively.

In the studied genes, we detected 7 rare protein coding variants: p.G2019S and p.R1441C (*LRRK2*), p.N409S, p.E365K, p.L483P and p.R535H (*GBA*) and p. A53T (*SNCA*).

*LRRK2* p.G2019S is the most common risk factor for sporadic PD ^16^ and together with p.R1441C clinically resembles late-onset, slowly progressive and L-DOPA responsive PD ^14,17^.

*SNCA* p.A53T causes early-onset, rapid progressive Parkinson’s disease.

*GBA* missense variants p.N409S, p.E365K, and p.L483P, are among the most significant PD risk factors and constitute more than 80% of *GBA* variants in the PD population. Overall, *GBA* variants in PD are associated with younger age at onset, rapid cognitive and motor progression, and a multitude of non-dopaminergic symptoms (i.e. postural instability, freezing of gait)^14^.

Of the 104 PD and prodromal patients, 41 (39%) carried pathogenic *LRRK2* mutations, with 39/41 (95%) being heterozygous carriers for *LRRK2* p.G2019S and 2/41 (5%) being heterozygous for *LRRK2* p.R1441C. Twenty-eight of the 104 patients (27%) carried pathogenic mutations in *GBA* in heterozygosity. Among these, 5 (18%) carried *GBA* p.E365K, 20 (71%) were heterozygous for p.N409S, 1 patient (3%) carried *GBA* p.E365K and N409S in compound heterozygosity, 1 patient (3%) carried *GBA* p.L483P and 1 patient (3%) was heterozygous for *GBA* p.R535H and *LRRK2* p.G2019S. A minority of patients 2/104 (2%) carried the autosomal dominant fully penetrant *SNCA* pathogenic mutation p.A53T in heterozygosity and 1/104 (1%) displayed a homozygous mutation in *LRRK2* p.G2019S. Thirty-seven patients (35%) did not carry any rare and known pathogenic mutation in *LRRK2*, *GBA* or *SNCA*. The majority of 104 PD and prodromal patients were homozygous for *APOE* ε3 (60%), 37 (35%) carried ε3 in heterozygosity (ε3/ε4 [22%] and ε2/ε3 [13%]). A minority of patients carried ε2/ε4 (2%) and one patient was homozygous for ε4. For one patient *APOE* genotype was not available.

Information regarding cardiovascular risk factors was available only for a minority of cases (at least 32/104 [31%]) and controls (18/48 [38%]). Among these, hyperlipidemia was the most common risk factor, detected in 66% of cases and 33% of controls. 40% of cases and 22% of controls displayed hypertension and 16% of cases and 5% of controls presented atrial fibrillation, respectively. 16% of controls and none of the cases had a valve prolapse or regurgitation and a minority of cases (5%) and none of the controls presented coronary artery disease, patent foramen ovale (PFO) or hypercoagulability (Table 1).

### Clinical features assessment

For the assessment of the clinical features, we used the Movement Disorder Society Unified Parkinson’s disease rating scale (MDS-UPDRS-III) and particularly for the basal ganglia symptoms such as akinesia and rigidity, the scores of the finger tapping and rigidity test, as previously described^18^. We used the Montreal Cognitive Assessment (MoCA) test for the cognitive function with a particular focus on executive and visuospatial function (trail making test, cube copy, or clock drawing) and memory impairment (delayed recall), which are critical in differentiating diverse dementing syndromes such as dementia with Lewy bodies, mostly associated with PD, and Alzheimer’s disease (AD), which frequently coexists with small vessel disease^19, 20, 21, 22^.

The clinical features (MDS-UPDRS-III and MoCA scores) were downloaded from the PPMI database (www.ppmi-info.org^10^.)

Among the clinical features of the cohort, PD patients presented an average MDS-UPDRS-III score of 28 (ranging from 5 to 64), prodromal patients an average score of 5 (ranging from 0 to 33) and the controls an average score of 4 (ranging from 0 to 4). Additionally, PD patients presented an average MoCA score of 26 (ranging from 9 to 30), the prodromal patients an average score of 28 (ranging from 20 to 30) and the controls an average score of 27 (ranging from 21 to 30).

For the PPMI cohort, the study has been approved by the WCG IRB on the 24th of July 2020. Ethical approvals and committees have been already described^10^.

The present study included patients from the PPMI cohort^10^. All methods were performed according to the relevant guidelines and regulations, including the Declaration of Helsinki and Good Clinical Practice^10^. Additionally, the study was authorized by regional ethics boards of the PPMI sites, all patients and controls taking part in the PPMI study submitted written informed consent. The PPMI study was approved by the institutional review board at each participating site. ^10^

The current analysis used only de-identified data from the PPMI database and was conducted according to the Declaration of Helsinki.

We confirm that we have read the Journal’s position on issues involved in ethical publication and affirm that this work is consistent with those guidelines.

### MRI acquisition

MRI images for healthy controls, familial PD and prodromal patients were acquired with the MP-RAGE axial T2-FLAIR MRI sequence (and when available axial T1-weighted on Siemens MRI scanners). MRI sequence information can be found in the MRI technical operations manual (www.ppmi-info.org*)*^23^.

It is a study requirement that the MRI scanner to be used for study acquisitions have a magnetic field strength of 3 Tesla. All scans for each participant must be acquired on the same scanner using the approved scanning protocol^10^.

All the subjects in our cohort received an axial T2-FLAIR MRI: 100/152 (66%) with a slice thickness of 5 mm (average of 30 total slices), 32/152 (21%) with a slice thickness of 4 mm (average of 33 total slices), 20/152 (13%) with a slice thickness of 3 mm (average of 49 total slices). 14/152 (9%) PD-Prodromal cases received an axial T2-FLAIR MRI follow-up and the average number of years between MRI scans was 4 (range 3–5 years)^10^.

### T2-FLAIR MRI scan analysis

All the MRI scans were separately rated twice by two experienced neurologists (H-M.S and V.K) and by two trained experienced residents in neurology (B.M and C.S). All raters were experienced in grading white matter abnormalities on MRI. Each scan was rated using the modified Scheltens scale^24^, representing a complement of the classical Fazekas scale, with the possibility of describing lobar distribution and rating cortical and subcortical white matter hyperintensities with a score (Table S3). Briefly, Scheltens and colleagues described 4 classes of white matter hyperintensities: periventricular hyperintensities, white matter hyperintensities, basal ganglia hyperintensities and infra-tentorial foci of hyperintensities.

We implemented this scale selecting 6 neuroradiological hallmarks for cerebral small vessel disease, based on the previous literature: periventricular hyperintensities^8^, lobar superficial white matter hyperintensities, ^25^, lobar deep white matter hyperintensities^8^, status cribrosus^26^, lacunar infarcts^9^ and lobar cortical small-microinfarcts^27^(Table S3, Fig. 1). Moreover, ischemic lesions such as cortical small-microinfarcts and lobar lacunes are a common feature of cerebral amyloid angiopathy (CAA)^28,27^ and were considered as CAA hallmark.

Our rating scale provides 7 sum scores in a semiquantitative way, as explained in Table S3: periventricular hyperintensities (PVH), lobar superficial white matter hyperintensities (LSWMH), lobar deep white matter hyperintensities (LDWMH), deep white matter hyperintensities, status cribrosus, lobar cortical small-microinfarcts, lacunar infarcts. PVH were identified as continuous, confluent areas of high signal intensity adjacent to anterior or posterior horns of the lateral ventricles (“caps”) and along the lateral ventricles (“bands”). LDWMH, located in the deep and subcortical white matter, were separately rated in the frontal, temporal, parietal and occipital regions; superficial white matter hyperintensities were defined as tracts originating within 5 mm of the cortical surface, as previously described^29^; status cribrosus describes the diffusely widened perivascular spaces (Virchow-Robin spaces) in the basal ganglia, especially in the corpus striatum on MRI^30,31^; cortical small and microinfarcts were defined as cortical hypointense lesions 15 mm and ≤ 4 mm in largest diameter respectively and distinct from perivascular spaces^27^; lacunar Infarcts were defined as lesions from 3 mm to < 15 mm in the supratentorial region, without cortical gray matter involvement^32^.

Manual segmentation on T2-FLAIR MRI images was conducted using the publicly available IMAIOS Atlas (https://www.imaios.com/en/e-Anatomy/Brain/Brain-MRI-in-axial-slices).

### Genetic study

#### CSVD patient cohort

All the patients included in the genetic study were Caucasian non-Hispanic from the US (NINDS [National Institute of Neurological Disorders and Stroke]). DNA was extracted and collected at the NINDS Repository. All NINDS Repository samples are collected only after a Coriell IRB-approved, signed informed consent is secured by the submitter. Biorepository projects at Coriell undergo annual IRB review and approval by the Coriell IRB.

The cSVD genetic study used only de-identified data from the NINDS Repository database and was conducted according to the Declaration of Helsinki.

Inclusion criteria comprised cerebral small vessel ischemic disease diagnosis based on TOAST classification, early age at onset (< 65 years [only 2 cases, whose age-at onset was 68 and 71 years have been included in the study because of a positive family history]), absence of known pathogenic mutations in Mendelian small vessel disease genes (*HTRA1*, *NOTCH3*, *ACTA2* and *COL4A1*) and no enrichment for vascular risk factors except for hypertension, which generally plays a critical role in elderly people^33^. The mean age at disease onset was 51.5 years (range 34–71 years). 82.3% of the cases were male and 44.8% of the cases were positive for a familial history of cerebrovascular disorders. Among the comorbidities and possible risk factors for cSVD, hypertension was reported in 60.4% of the patients, type 2 diabetes in 30.2%, and myocardial infarction in 7.3%. The majority of the patients (at least 88.54%) were negative for atrial fibrillation (AF), which is among the most important risk factors for embolic small vessel occlusion^34^. In 4.1% and 7.3% of the patients the presence of AF was reported and unknown, respectively. Given the prevalent role of hypertension and type 2 diabetes in cSVD in the elderly people^33^ and the young age at onset of the cohort, these patients were considered enriched for genetic risk factors (Table 2). Finally, 243 controls > 80 years of age were selected from ‘HEALTHY EXOMES’, HEX, a publicly available database, which collects exome sequencing data from elderly neuropathologically proven controls (https://www.alzforum.org/exomes/hex;^35^).

**Table 2:** cSVD and CTRLS Exome Sequencing cohort

Country of origin	*N*	Disease	AAO (SD)/Age	M: F	*N* familial cases (%)	Hypertension (%)	Diabetes (%)	Migraine (%)	Heart comorbidities (MI, AF) (%)	Hypercholesterolemia (%)
US	96	cSVD	51.5 (8.1)y	0.82	43 (45)	58 (60)	29 (30)	NA	11 (11)	2 (2)
GB	243	CTRLS	¬ 80y		NA	NA	NA	NA	NA	NA

cSVD, cerebral small vessel ischemic disease; AAO, age at onset; SD, standard deviation; M, male; F, female. MI, myocardial infarction; AF, atrial fibrillation; y, years; GB, Great Britain; NA, not available.

The experimental protocols were approved by the PPI licensing committee^10^.

For both the NINDS and PPMI cohorts informed consent was obtained from all subjects and/or their legal guardian(s).

### Exome sequencing

We performed whole-exome sequencing on a cohort of 96 independent familial and early-onset apparently sporadic cSVD cases. DNA was extracted from blood using standard protocols: genomic DNA was purified from fresh blood using the Qiagen Autopure LS instrument according to the manufacturer’s instructions (whole blood). Briefly, cells from whole blood were lysed by addition of anionic detergent, and RNA and Protein were degraded with the addition of RNase and Proteinase K. After mixing, Autopure Precipitation Solution (Autopure) was added, and the insoluble cell debris was removed by centrifugation. An equal volume of isopropanol (Autopure) was added to the supernatant, and the resulting DNA precipitate was collected by centrifugation (Autopure). Following a brief rinse with 70% ethanol to remove residual salt, the DNA pellet was solubilized in TE Buffer (10 mM Tris, pH 8.0/l mM EDTA).

Library preparation for next-generation sequencing used 50 ng of DNA. Exome libraries were prepared using Nextera Rapid Capture Exome Kit (4 rxn × 12 plex, FC-140-1002). The DNA library was then hybridized to an exome capture library (Nextera, Illumina) and precipitated using streptavidin-coated magnetic beads (Nextera, Illumina). Exome-enriched libraries were PCR-amplified, and then DNA was hybridized to paired-end flow cells using a cBot (Illumina) cluster generation system. Samples were sequenced on the Illumina HiSeq 3000/4000 using 2 × 76 paired end reads cycles, as previously described^37^.

### Bioinformatics, exome sequencing

The reads were aligned using BWA-MEM v0.7.15 to the reference GRCh37 (hs37d5.fa), separate read groups were assigned for all reads from one lane, and duplicates were masked using Samblaster v0.1.24^38^. Standard QC was performed using FastQC (http://www.bioinformatics.babraham.ac.uk/projects/fastqc). The variants were then called using GATK UnifiedGenotyper v3.7^39^ and annotated using Jannovar v0.24^40^ using RefSeq v105 exons. We used Clinvar database (https://www.ncbi.nlm.nih.gov/clinvar/) to assess the potential pathogenicity of single variants, as already reported^41^. Moreover, variants were re-interpreted based on the American College of Medical Genetics and Genomics (ACMG) criteria^42^.

All rare missense variants within the coding regions of 11 Mendelian or genome-wide association study (GWAS) PD genes (*VPS35* [ENSG00000069329], *DJ1* [ENSG00000116288], *PINK1* [ENSG00000158828], *ATP13A2* [ENSG00000159363], *PRKN* [ENSG00000185345], *SNCA* [ENSG00000145335], *LRRK2* [ENSG00000188906], *GBA* [ENSG00000177628], *MAPT* [ENSG00000186868], *LAMP3* [ENSG00000078081], *STK39* [ENSG00000198648] have been collected and analysed.

### Statistical analysis and methods to prevent bias

This is an exploratory, descriptive study. Sample sizes were not based on a priori power calculation.

For the exome sequencing and the MRI PPMI cohorts, post hoc power calculations were performed with R statmod-package v1.4.32 and Gpower Software (http://www.gpower.hhu.de/) .

To compare our results we have used Wilcoxon-Test and Fisher’s exact Test in R version 3.3.2. Ggplot2 in R was used to draw the graphs.

Spearman’s correlation analysis and linear mixed-effects analysis were performed with jamovi (https://www.jamovi.org/).

The Exome Sequencing Analysis had 80% power for the detection of common variants MAF > 5% with strong effect (OR < 0.6 or > 2), with a significance value of two-sided α = 0.05. Low frequency and rare variants were defined as having a 1%< MAF < 5% and MAF < 1%, respectively, either in cases or controls. Minor allele frequency was based either on HEX database for elderly controls > 70 years of age or ExAC database version 0.3.1 database (http://exac.broadinstitute.org/), as already described^41^.

The MRI PPMI Study had 80% power for the detection of white matter lesions of medium effect size (d = 0.5), with a significance value of two-sided α = 0.05.

All methods were carried out in accordance with the relevant guidelines and regulations.

## Results

### CSVD neuroimaging hallmarks in familial PD and control patients

To investigate the hypothesis that familial PD and cSVD may have shared pathogenic mechanisms we used a modified Scheltens Scale (Table S3) and performed an exploratory study of the main 7 cSVD neuroradiological hallmarks (periventricular hyperintensities, lobar superficial white matter hyperintensities, lobar deep white matter hyperintensities, deep white matter hyperintensities, status cribrosus, lobar cortical small-microinfarcts, lacunar Infarcts)^8,9^ in the T2-FLAIR MRI sequences in a cohort of 58 familial PD and 46 familial prodromal PD patients and 48 age-matched controls from the PPMI database (Fig. 1).

Importantly, 7 controls (14%, average age 55y, range: 32y-71y), 4 PD prodromal patients (9%, average age 58y, range: 51y-67y) and 1 PD patient (2%, age 69y) did not display any white matter hyperintensity, were associated with no signs of cognitive impairment (MoCA average 30) and only with subtle motor impairment (MDS-UPDRS-III, average 4, ranging from 9 − 1). Among these, the PD patient did not carry any rare known pathogenic mutation in *GBA*, *LRRK2* and *SNCA* and among the PD prodromal patients 3/4 (75%) and 1 carried *LRRK2* p.G2019S and *GBA* p.N409S, respectively.

### Single cSVD neuroradiological biomarker analysis

**Fig. 2:**
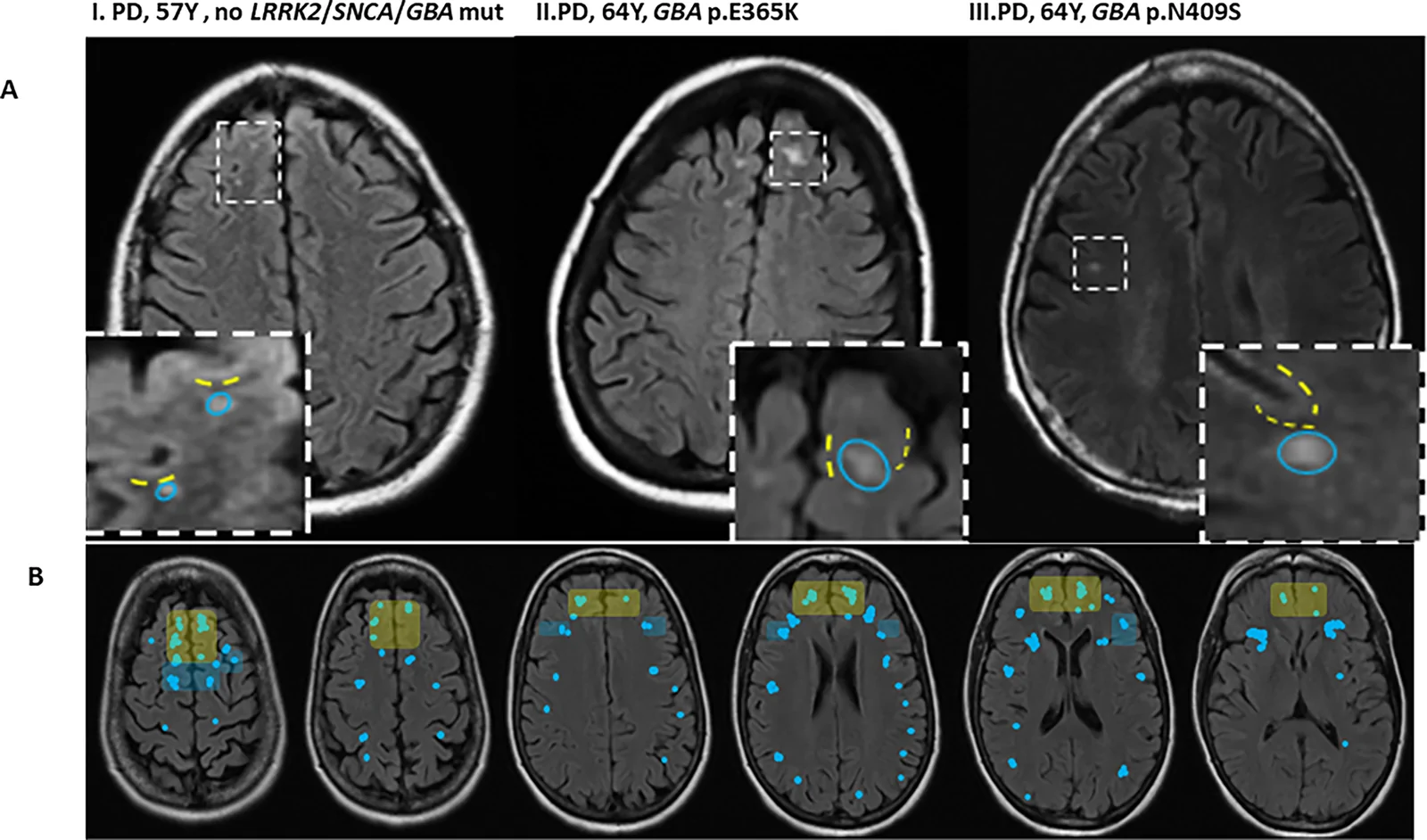

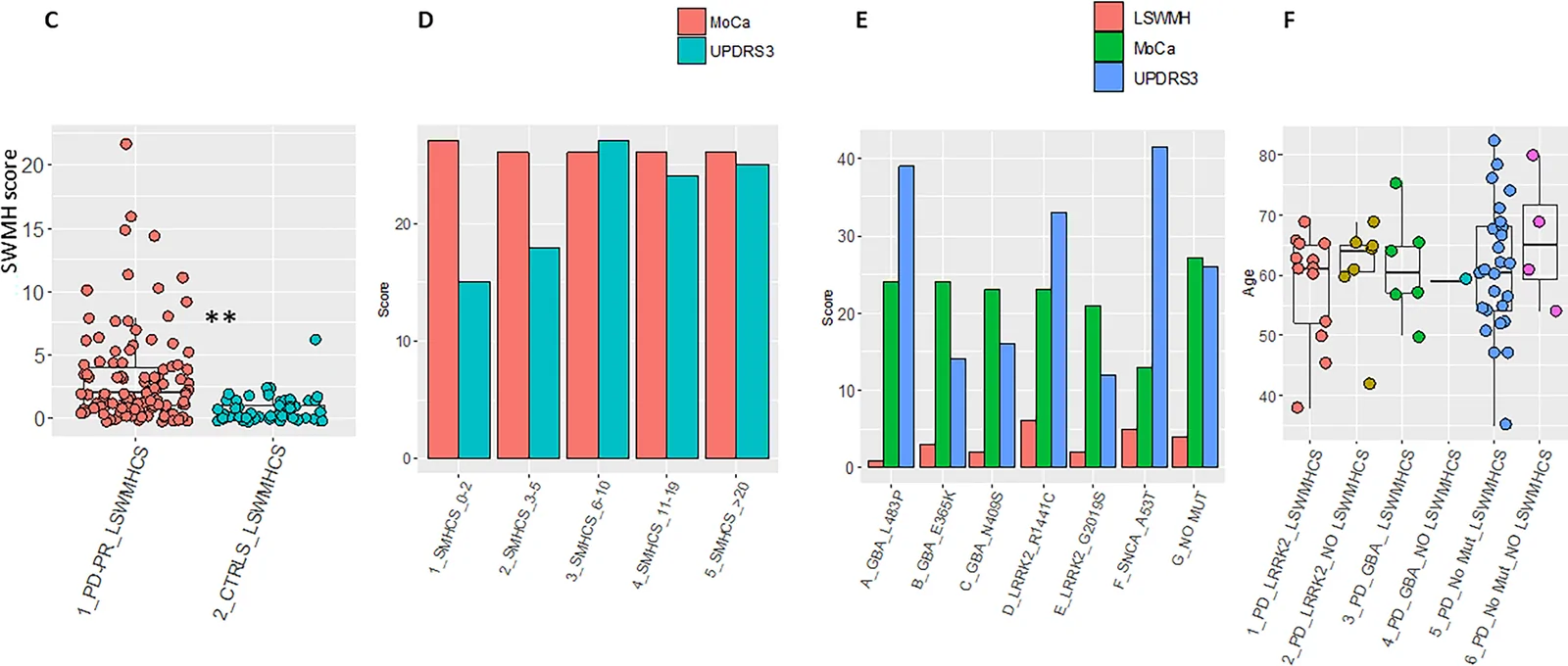
Superficial white matter hyperintensities in PD and prodromal PD patients particularly clustering in the superior frontal gyrus and framed within a dashed line (2 A I, II, III). **AI** PD patient, 57 years old, not carrying any rare known pathogenic mutation in *LRRK2*, *GBA* and *SNCA*, homozygous for *APOE* ε3 allele, presenting a severe motor impairment (MDS-UPDRS-III 31) and no cognitive deficits (MoCA 27) and a LSWMH score of 10. **A II**, PD patient, 64 years old, carrying *GBA* p.E365K, homozygous for *APOE* ε3 allele, presenting a mild motor impairment (MDS-UPDRS-III 16) and mild cognitive impairment (MoCA 25) and LSWMH score of 11. **A III**, PD patient, 64 years old, presenting *GBA* p.N409S homozygous for *APOE* ε3, displaying no motor impairment (MDS-UPDRS-III 0) and no cognitive deficits (MoCA 27), LSWMH score of 3. **2B** Collective representation of the superficial white matter hyperintensities in the 104 PD and prodromal PD patients analysed in the study, clustering in the frontal superior and to a lesser extent inferior gyrus, corresponding to the Brodmann area 8 (in yellow) and Brodmann area 6 (in light blue). Each blue dot represents a single superficial white matter hyperintensity.**2C** Familial PD patients and familial PD prodromal patients displayed a statistically significant burden of cortical frontal superficial white matter hyperintensities (p-value = 3.46e-06, Bonferroni-corrected). PD, familial Parkinson’s disease patients; PR, familial prodromal Parkinson’s disease patients; CTRLS, controls; **, p-value  <0.00005. **2D** Bar plot showing an increasing superficial white matter cumulative score and a parallel progressive subtle decrease of MoCA scores and marked increase of MDS-UPDRS-III scores. **2E** Bar plot describing a mild to very severe dementia caused by *GBA*, *LRRK2* and particularly *SNCA* mutations and a concomitant increase of MDS-UPDRS-III motor scores, paralleled by an enrichment in superficial lobar white matter hyperintensities particularly in *LRRK2* p.R1441C and *SNCA* p.A53T. **2 F**, Plot describing an earlier age at onset in PD patients carrying *LRRK2* variants and PD patients not carrying known pathogenic *LRRK2*, *GBA* and *SNCA* variants with LSWMH compared to PD patients carrying the same genetic variants and not presenting LSWMH. LSWMH, lobar superficial white matter hyperintensity; WMHCS, white matter hyperintensity cumulative score.

Analyzing individual neuroradiological cSVD biomarkers, we found a statistically significant increased burden of superficial white matter hyperintensities in familial PD-prodromal and PD patients (81/104, 78%) compared to age-matched controls (22/48, 46%) (Wilcox Test p-value = 3.46e-06, Bonferroni-corrected) (Fig. 2A–C).

Furthermore, 19% and 6.5% of familial PD and PD prodromal patients, respectively, presented several (> 6) superficial white matter hyperintensities and more than 10% of the patients presented lesions in multiple lobes, affecting in > 60% of these cases the frontal lobe (Fig. 2A, B). Importantly, patients carrying *LRRK2* p.R1441C presented an average LSWMH score of 6 (range: 4–8), followed by patients carrying *SNCA* p.A53T with average LSWMH score of 5.5 (range:3–8) and prodromal-PD patients not carrying known pathogenic mutations in *LRRK2*, *GBA* and *SNCA*, displaying an average LSWMH of 4 (range: 0–22)(Fig. 2E).

Interestingly, PD patients carrying SWMH and *LRRK2* p. R1441C and p. G2019S displayed an earlier age at onset compared to patients carrying the same mutations but not presenting SWMH (average age at onset 58y vs. 61y, respectively) (Fig. 2F).

Superficial white matter lesions clustered mostly in the superior frontal and inferior frontal gyri, encompassing the Brodmann areas 6 and 8 (Fig. 2A-B).

The increase of superficial white matter hyperintensity score was paralleled by a mild increase of the MDS-UPDRS-III score (from 14 to 25). This trend was characterized by a subtle accentuation of bradykinesia and gait impairment, consistent with observations already reported in PD ^36^ and an average modest decrease in the MoCA score (from 28 to 26) particularly affecting visuospatial orientation, attention, verbal fluency and memory, however, these scores remained within the range of normal cognitive functioning(Fig. 2D).

**Fig. 3:**
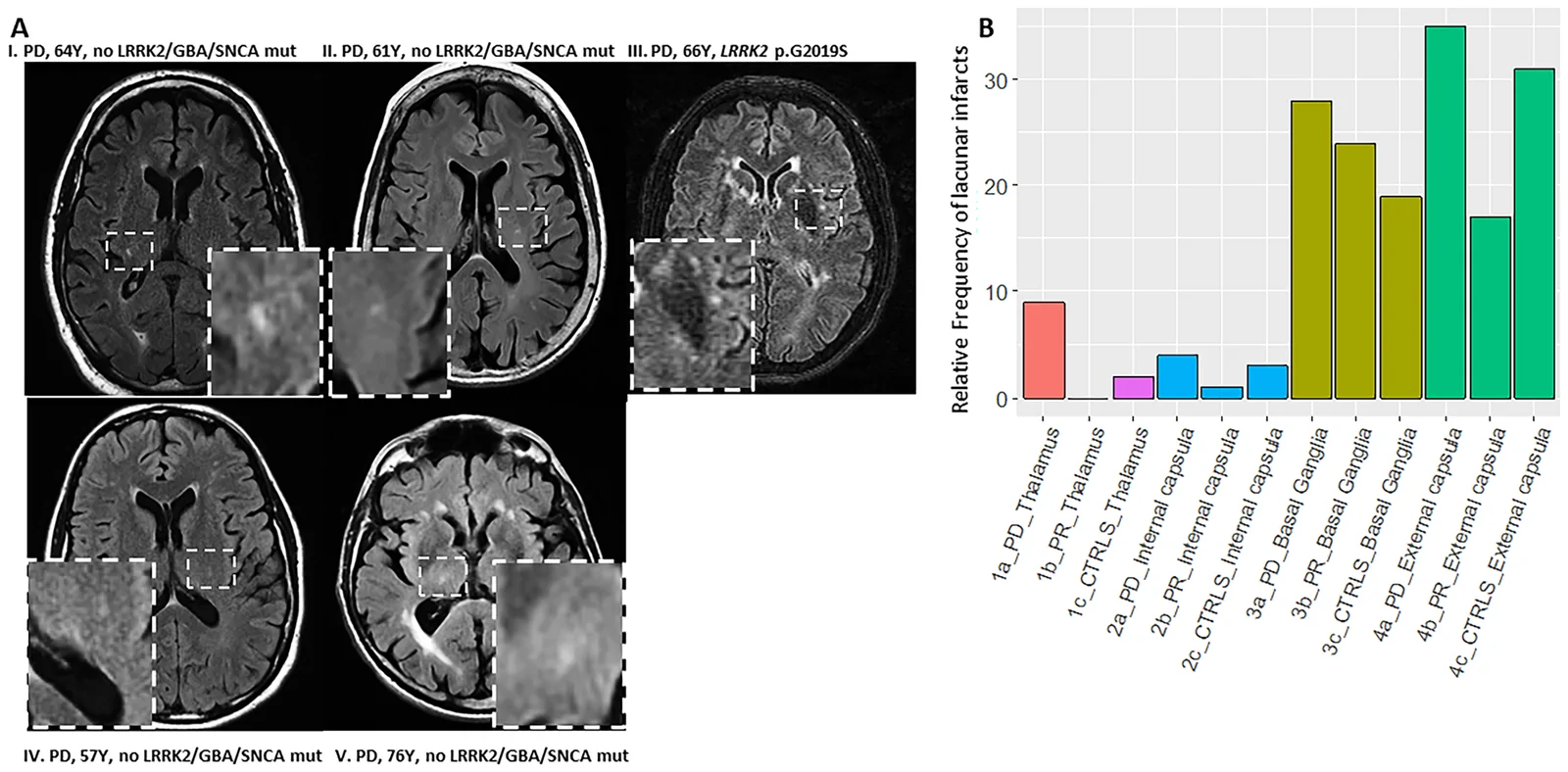
A **I**-**V** Axial T2-FLAIR MRI scans showing lacunar infarcts detected in 5 late-onset familial PD patients, framed within a dashed line. **I** Right thalamus infarct in a PD patient, 64 years old, not carrying any rare known pathogenic mutation in *LRRK2*, *GBA* and *SNCA*, heterozygous for *APOE* ε3 (ε2/ ε3), presenting a mild motor impairment (MDS-UPDRS-III 13) and mild cognitive impairment (MoCA 24). **II**, Left corona radiata infarct in a PD patient, 61 years old, not carrying any known rare pathogenic mutation in *LRRK2*, *GBA* and *SNCA*, heterozygous for *APOE* ε3 (ε2/ ε3), presenting a mild motor impairment (MDS-UPDRS-III 17) and no signs of dementia (MoCA 27). **III** Left capsula externa infarct in a PD patient, 66 years old, carrying *LRRK2* p.G2019S, homozygous for *APOE* ε3, presenting a severe motor impairment (MDS-UPDRS-III 48) and no cognitive impairment (MoCA 28). **IV**, Left thalamus infarct in a PD patient, 57 years old, not carrying any rare known pathogenic mutation in *LRRK2*, *GBA* and *SNCA*, homozygous for *APOE* ε3, presenting a severe motor impairment (MDS-UPDRS-III 31) and no cognitive deficits (MoCA 27). **V**, Right thalamus infarct in a PD patient, 76 years old, not carrying any rare known pathogenic mutation in *LRRK2*, *GBA* and *SNCA*, heterozygous for *APOE* ε4 (ε3/ ε4), presenting a mild motor impairment (MDS-UPDRS-III 18) and no cognitive deficits (MoCA 30) **B.** Relative frequency of different lacunar infarcts (thalamus, basal ganglia, capsula interna and capsula externa) detected in our cohort. PD, familial Parkinson’s disease patients; PR, familial prodromal Parkinson’s disease patients; CTRLS, controls.

In addition, multiple bilateral lacunar infarcts in the basal ganglia, capsula interna and externa were detected both in elderly cases (familial PD patients [14/58, 24%], prodromal patients [9/46, 19%]) and controls (14/48, 29%) without any statistically significant difference (p-value = 0.41, 95% CI =-0.29-1.63). However, 30% of familial PD patients and particularly PD patients carrying *LRRK2* p.G2019S and p.R1441C mutations presented a burden of lacunar strokes clustering in the thalamus (relative frequency 9% in familial PD cases and 2% in age-matched controls, p-value = 0.058), which appeared to align with a hypokinetic-rigid phenotype, featuring typical basal ganglia signs such as moderate akinesia and rigidity (MDS-UPDRS-III average score 3 for finger tapping and 4 for rigidity of extremities, respectively)^18^(Fig. 3A-B).

**Fig. 4:**
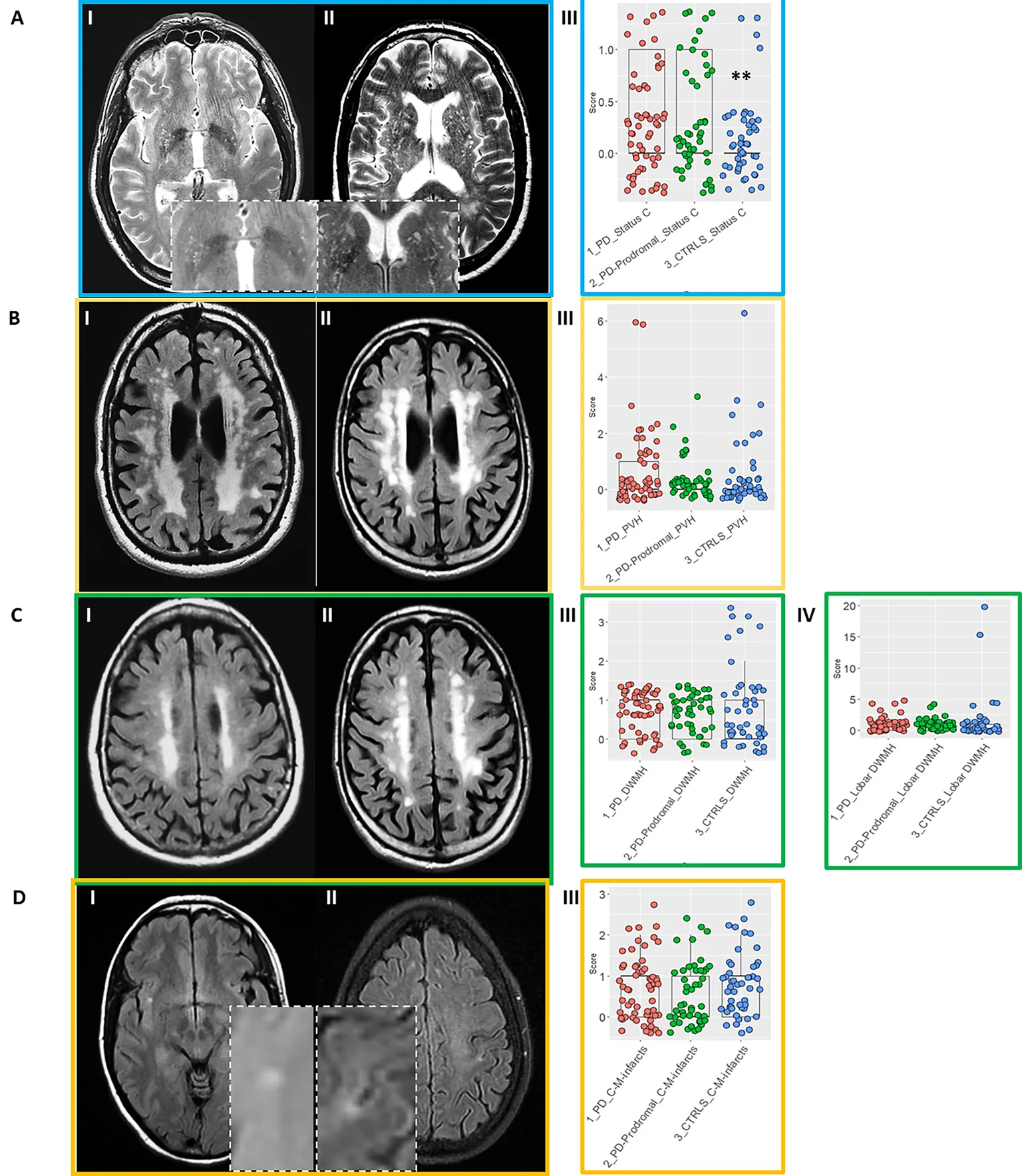
**A-D**. Axial T2-FLAIR MRI scans displaying enlarged perivascular spaces, periventricular white matter hyperintensities, deep white matter hyperintensities, cortical microinfarcts and corresponding box-dot-plots of PD and PD prodromal patients and controls. **A**. **Enlarged perivascular spaces**. **I** Control, 74 years old, homozygous for *APOE* ε3, with a very mild motor impairment (MDS-UPDRS-III 3), and subtle cognitive impairment (MoCA 25). **II** PD patient, 71 years old, not carrying any rare known pathogenic mutation in *LRRK2*, *GBA* and *SNCA*, heterozygous for *APOE* ε4 (ε3/ε4), presenting a severe motor impairment (MDS-UPDRS-III 31) and mild cognitive impairment (MoCA 20). **III** Box-dot plot displaying a significant enrichment of basal ganglia enlarged perivascular spaces in PD and PD prodromal patients (p-value = 2.64e-03, Bonferroni-corrected) compared to controls. ^**^ p-value < 0,005. **B** **Periventricular white matter hyperintensities**. **I** Control, 81 years old, homozygous for *APOE* ε3, presenting a severe motor impairment (MDS-UPDRS-III 31) and mild cognitive impairment (MoCA 23). **II** PD patient, 76 years old, heterozygous for *APOE* ε4 (ε3/ε4), displaying a mild motor impairment (MDS-UPDRS-III 18) and no cognitive deficits (MoCA 30). **III** Box-dot plot presenting a similar degree of periventricular white matter hyperintensities between PD and PD prodromal patients and controls. **C** **Deep white matter hyperintensities**. **I** Control, 81 years old, homozygous for *APOE* ε3, with very subtle motor impairment and cognitive deficit (MDS-UPDRS-III 3 and MoCA 27). **II** PD, 76 years old, heterozygous for *APOE* ε4 (ε3/ε4), presenting a mild motor impairment (MDS-UPDRS-III18) and no cognitive deficits (MoCA 30). **III-IV** Box-dot plot presenting overlapping deep white matter hyperintensity severity between PD and PD prodromal patients and controls. **D Cortical microinfarcts**. **I** Control, 43 years old, homozygous for *APOE* ε3, with no motor deficits (MDS-UPDRS-III 0) and subtle cognitive impairment (MoCA 26). **II** PD patient, 60 years old, carrying *LRRK2* p.G2019S, homozygous for *APOE* ε3, with severe motor impairment (MDS-UPDRS-III 42) and no cognitive deficits (MoCA 27). **III** Box-Dot plot showing no cortical microinfarct significant difference between PD and PD prodromal patients and controls.

Moreover, a mild, bilateral enlargement of the basal ganglia perivascular spaces particularly affecting the nucleus lentiform to a severe status cribrosus was found in 31/104 familial PD and prodromal cases (30%, average age 62y [range:38y-82y] ) and only in 4/48 (8%, average age 69 y[range: 56y-81y]) controls (p-value = 2.64e-03, Bonferroni-corrected) (Fig. 4A I-III). Among the PD-Prodromal cases 13/31 (42%) carried *LRRK2* p.G2019S and 11/31 (35%) did not carry any rare known pathogenic mutation in *LRRK2*, *SNCA* and *GBA*.

Furthermore, we did not report any significant phenotypic correlation in terms of age at onset (average 61y), MDS-UPDRS-III and MoCA Scores (18 and 27, respectively) between different degrees of perivascular space enlargement to a severe status cribrosus in PD and familial PD prodromal patients and controls. However, as reported in previous studies, PD patients exhibited a tendency toward a mild rigidity, notably of the neck and upper extremities^43^.

We did not identify any lacunar infarct in the pons or mesencephalon.

On the contrary, PD and PD prodromal cases did not present any substantial difference in periventricular hyperintensities, lobar deep white matter hyperintensities, deep white matter hyperintensities and cortical small-microinfarcts when comparing individuals with PD to controls (p-value ≥ 0.05) (Fig. 4B-D).

### Cumulative cSVD neuroradiological biomarker analysis

**Fig. 5:**
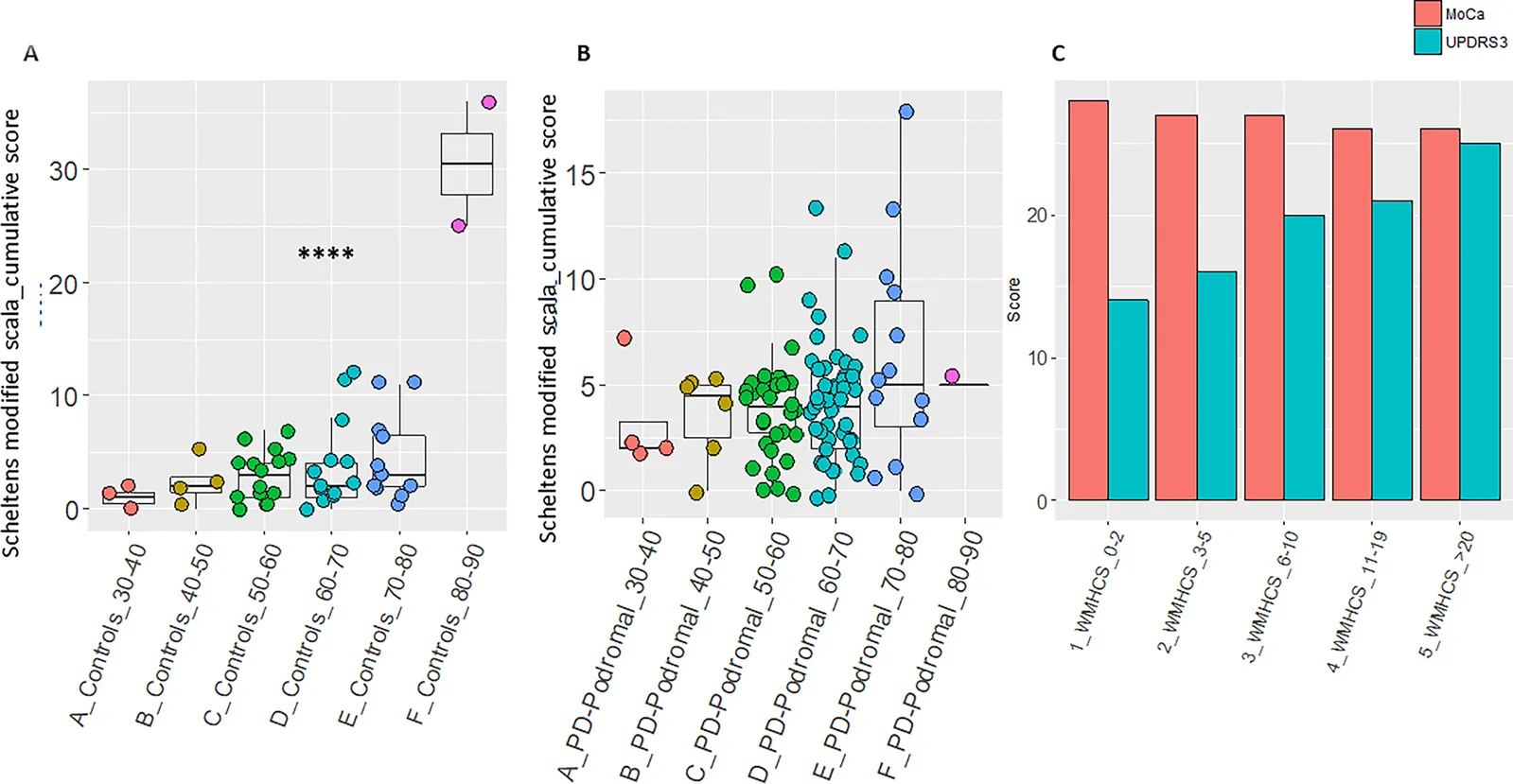
White matter hyperintensity cumulative score obtained from the sum of the 7 cSVD neuroradiological hallmarks, showing a strong, linear and age-dependent increase, both for controls (**A**) and PD and prodromal PD patients (**B**). **C** Bar plot showing an increasing white matter hyperintensity cumulative score and a parallel progressive subtle decrease of MoCA scores and marked increase of MDS-UPDRS-III scores. **** p-value  <0.00005.

When considering the cSVD neuroradiological biomarker cumulative score, we found no statistically significant difference between familial PD patients, familial prodromal PD patients and controls (p-value = 0.53, 95% CI -1.95-1.02). We report a linear and age-dependent increase of the cSVD neuroradiological hallmark cumulative score, mostly driven by superficial lobar white matter hyperintensities both for familial PD, PD prodromal patients and for controls, reaching in the latter ones a statistical significance (p-value = 6.0e-06, Bonferroni-corrected) and more marked in the frontal lobe, representing 45% of the hemisphere^27^(Fig. 5A-C, Table S5).

Moreover, in our cohort we did not find any significant difference in terms of white matter hyperintensity cumulative score in PD-PD prodromal patients carrying cardiovascular risk factors (type II diabetes, hypertension, hyperlipidemia, hypercholesterolemia, atrial fibrillation, coronary artery disease, pre-diabetes, hypertensive heart disease, carotid atheroma, pervio ovale foramen) and PD-PD prodromal patients not carrying any vascular risk factor (WMHCS 5.5 and 5.7, respectively). None of the PD-PD prodromal patients with known cardiovascular risk factors was a smoker (Table S5).

### Longitudinal MRI and clinical follow-up

Longitudinal MRI follow-up data were available only for a minority of PD- and PD prodromal patients: 14 PD-PD Prodromal patients (13%, 1 PD and 13 PD-prodromal patients). The average time between MRI scans was 4 years (range 3–5 years).

A mild increase of WMHCS (average of 3 [range 0–15] and particularly pronounced in the single PD patient) corresponded to a proportional increase in MDS-UPDRS-III scores (average 4 [range 0–14] and particularly in the PD patient). By contrast, MoCA scores remained mostly stable between MRI scans (average MoCA score difference of 0.4 [range 0–6])(Table S6 and Figure S1).

To assess whether progression of cSVD burden was significantly associated with clinical progression, we examined the association between ΔWMHCS and changes in MoCA and MDS-UPDRS-III scores using Spearman correlation analyses and linear mixed-effects models.

Spearman correlation analyses showed no significant association between ΔWMHCS and ΔMoCA (ρ = -0.425, *p* = 0.130) neither between ΔWMHCS and ΔUPDRS III (ρ = 0.175, *p* = 0.551). In linear mixed-effects models including subject-specific random intercepts, the interaction between time and ΔWMHCS was not significantly associated with MoCA scores (β = -0.053, 95% CI -0.117 to 0.011, *p* = 0.106). Additionally, UPDRS III score progression was significantly associated to ΔWMHCS in the full model (β = 0.125, 95% CI 0.026 to 0.223, *p* = 0.013). However, this association was not retained after excluding one patient with marked WMHCS progression (ΔWMHCS = 15; β = 0.022, 95% CI -0.170 to 0.213, *p* = 0.823), suggesting that the motor association was driven by a single outlier.

Overall, these longitudinal analyses do not provide robust evidence that progression of WMHCS burden is consistently associated with cognitive nor motor impairment. However, given the small sample size, we may not exclude a positive significant association in larger follow-up PD-PD prodromal patient cohorts and/or over a longer period of time.

### *LRRK2*, *GBA* and *SNCA* diverse endophenotypes in the PD and PD prodromal cohort

Monogenic and oligogenic PD forms displaying heterozygous *LRRK2*, *GBA* variants with variable penetrance, were characterized by a pleomorphic phenotype and generally they resembled the sporadic form of the disease: presented an older age at onset (average 62y), milder motor and cognitive phenotype (average MDS-UPDRS-III 12 and MoCA 27) and mild WMHCS (average 6), mostly driven by superficial lobar white matter hyperintensities (Table S4 and S5, Fig. 2E). Among these, a mild cognitive impairment (25 ≥ MoCA score ≥ 18) was detected in 6/39 (15%) of *LRRK2* p.G2019S carriers, and particularly in homozygous state linked to a significant motor impairment (MDS-UPDRS-III 29). Analogously, *LRRK2* p.R1441C carriers displayed a mild cognitive impairment and severe motor impairment (MoCA 23 and MDS-UPDRS-III 33). Additionally, 7/28 (25%) carriers of *GBA* coding variants (p.N409S, p.E365K, p.L483P and p.R535H) suffered a mild cognitive deficit (MoCA average 23) and only 4/27 (15%) *GBA* mutation carriers were characterized by very severe motor impairment (MDS-UPDRS-III 39) (Table S4 and S5, Fig. 2E).

By contrast, PD patients presenting a monogenic form, carrying the autosomal dominant fully penetrant *SNCA* p.A53T mutation, presented a very early onset (32y and 53y) and were characterized by very severe dementia (MoCA score 17 and 9, respectively) and severe motor impairment (MDS-UPDRS-III 37 and 46, respectively). This phenotype was only minimally influenced by cerebral white matter hyperintensities (WMHCS 10 and 6, respectively and particularly superficial lobar white matter hyperintensities) (Table S4 and S5, Fig. 2E).

Finally, *APOE ε2* allele has been associated with increased severity of small vessel disease^44,45^ however, we have not detected any statistically significant difference between PD and prodromal PD patients and controls as in our cohort 16/104 (15%) patients and 4/48 (8%) controls carried this allele in heterozygosity (p-value = 0.3059, 95% CI = 0.59–8.67, OR 1.9)(Table 1, Table S4).

### PD Mendelian genes and main GWAS loci genetic screening in familial cSVD patients

To further investigate the hypothesis that PD Mendelian genes and main common risk factors may be associated with cSVD, we screened protein coding variability in 11 PD causative genes (*VPS35*, *DJ1*, *PINK1*, *ATP13A2*, *PRKN*, *SNCA*, *LRRK2*) and genetic risk factors (*LRRK2*,* GBA*, *MAPT*, *LAMP3*, *STK39*) in 96 early-onset unrelated cerebral small-vessel disease cases and 243 elderly controls neuropathologically proven, from the UK (Table 2). We did not identify any rare known pathogenic variant in the studied genes in our cSVD cohort (Table 3, Table S7).

**Table 3:** Rare coding variants in PD Mendelian genes and PD main Genome Wide Association Study (GWAS) Loci detected in our discovery cohort

Gene	Inh.	Position	rsID	Ref/Alt	cDNA	Aa	Gen	ExAC	ACMG classification	ClinVar		SVD (%)	Gender	AAO	PD	Family history	Vascular risk factors
*ATP13A2*	AR	chr1: 17,323,565	rs756955409	T/C	c.1130 A > G	p.Q377R	Het	0.0	UNK [PM2,PP3]	Not present		1/96 (1)	F	60y	no	absent	Smoking (40 years)
*ATP13A2*	AR	chr1: 17,323,581	rs886395233	C/T	c.1114G > A	p.G372R	Het	0.0	Likely pathogenic [PP3(Strong), PM2]	Not present		1/96 (1)	M	49y	no	absent	Former smoker (34 years)
*PINK1*	AR	chr1: 20,971,032	rs778009684	C/T	c.826 C > T	p.R276W	Het	4.118e-05	UNK [PM2,PP3(Supporting)]	Uncertain significance		1/96 (1)	M	48y	no	present (father with MI at 77y)	Positive familial history
*PINK1*	AR	chr1: 20,972,069	rs376323248	C/T	c.976 C > T	p.R326C	Het	1.647e-05	UNK[PM2]	Not present		1/96 (1)	M	51y	no	absent	Smoking (35y), hypertension
*SNCA*	AD	chr4: 90,650,386	rs145138372	G/T	c.349 C > A	p.P117T	Het	4.118e-05	UNK[PM2]	Uncertain significance		1/96 (1)	M	42y	no	Present (mother, CVA)	hypertension
*PRKN*	AR	chr6: 161,771,240	rs191486604	C/T	c.1289G > A	p.G430D	Het	9.885e-05	Pathogenic[PP5(Strong), PP3(Strong), PM1(Strong)]	Pathogenic/Likely pathogenic (in homozygosity)		1/96 (1)	M	41y	no	Present (Grandmother)	Hypertension treated with medications
*PRKN*	AR	chr6: 161,771,243	rs760223151	C/T	c.1286G > A	p.G429E	Het	1.648e-05	Likely Pathogenic [PM1(Strong), PM2,PP3]	Not present		1/96 (1)	F	62y	no	Present (mother)	Smoking (25y),
*LRRK2*	AD, risk factor	chr12: 40,619,054	rs1422672946	G/T	c.121G > T	p.D41Y	Het	0.0	UNK[PM2]	Not present		1/96 (1)	M	42y	no	absent	Smoking (25 years), hypertension treated with medications
*LRRK2*	AD, risk factor	chr12: 40,631,833	rs1465061766	A/T	c.499 A > T	p.M167L	Het	0.0	UNK[PM2,BP4(supporting)]	Not present		1/96 (1)	M	64y	no	absent	Former smoker
*LRRK2*	AD, risk factor	chr12: 40,713,943	rs766206415	C/A	c.4981 C > A	p.P1661T	Het	4.119e-05	UNK[PM2]	Not present		1/96 (1)	M	43y	no	Present (mother)	Current smoker (21 years)

Ref, reference base; Alt, alternative base; Aa, amino-acid; SVD, small vessel ischemic disease; AD, autosomal dominant, AR, autosomal recessive; AAO, age-at onset; PD, Parkinson’s disease; y, years. Ihn., inheritance; ACMG, American College of Medical genetics and genomics.

None of the detected variants in heterozygosity has been reported as pathogenic or likely pathogenic in ClinVar database (https://www.ncbi.nlm.nih.gov/clinvar).

Six variants (*ATP13A2* p.Q377R and p.G372R, *PINK1* p.R326C, *LRRK2* p.D41Y, p.M167L and p.P1661T) are not present in the ClinVar database. Additionally, *PRKN* p.G430D was found in heterozygosity in a male patient with very early-onset (41y), familial cSVD with a positive familial history (grand-mother affected) and hypertension treated with medications. Importantly, *PRKN* p.G430D in homozygosity causes autosomal recessive juvenile PD whereas in heterozygosity it has not been linked to increased risk for PD^46^.

Overall, elderly controls displayed a higher relative frequency of variants in the studied genes (Figure S2). The only exception were coding variants in *SNCA*, presenting a relative frequency of 1% (1/96) and 0.4% (1/243) in cSVD patients and elderly controls, respectively.

Moreover, 10/96 (10%) early-onset cSVD cases and 82/243 (34%) elderly controls carried at least 1 rare coding variant in the studied genes. All the variants detected in the cSVD cohort were singletons. None of the cSVD patients carried any variant in homozygosity or compound heterozygosity. In addition, in these cSVD cases we have not identified any rare coding variants in *GBA*, *LAMP3*, *MAPT*, *DJ1*, *STK39* and *VPS35*. We detected a total of 9 rare coding variants in *ATP13A2* (p.G372R, p.Q377R), *PINK1* (p.R276W, p.R326C), *SNCA* (p.P117T), *PRKN* (p.G429E) and *LRRK2* (p.D41Y, p.M167L, p.P1661T), absent in controls (Table 3 and Table S7).

*ATP13A2* and *PRKN*, *PINK1* and *SNCA* harbor the lowest and highest relative frequency of low-frequency and rare coding variants (mean 0.5 and 0.3 low-frequency-rare variants per kb of coding sequence, respectively). 90% of the variants were described as probably-damaging or possibly-damaging by in-silico prediction software (PolyPhen2).

Additionally, *LRRK2* p.G2019S, the most common cause of familial PD (5–6%) and a risk factor for sporadic PD (1%)^47^ was detected with a frequency of 4e-4 in the elderly controls and in none of the cSVD familial cases (Table 3 and Table S7).

## Discussion

Using neuroradiology and genetics, we explored the hypothesis that familial PD, VP, and its hallmark, cSVD, which exhibit varying degrees of overlapping phenotype, may coexist, influence each other and may be linked by a shared pathophysiology. To this end, we selected a cohort of 104 familial PD and familial PD prodromal patients from the PPMI database and used a modified Scheltens Scale to screen T2-FLAIR MRI sequences for the main cSVD neuroradiological biomarkers (i.e., periventricular hyperintensities, lobar superficial white matter hyperintensities, lobar deep white matter hyperintensities, deep white matter hyperintensities, status cribrosus, lobar cortical small-microinfarcts, lacunar Infarcts) (Fig. 1).

Importantly, in our cohort, PD patients tended to display a burden of white matter hyperintensities encompassing the lobar superficial layers to the basal ganglia (Figs. 2, 3 and 4).

Our exploratory study pointed to an enrichment for superficial white matter hyperintensities in the frontal superior and inferior gyri and to a lesser extent in the parietal lobe, mainly corresponding to the Brodmann area 8 and to the premotor cortex (Brodmann area 6), in PD and PD prodromal patients, particularly in carriers of *LRRK2* p.R1441C and *SNCA* p.A53T (Fig. 2A-B and E). These superficial white matter lesions could potentially contribute to microstructural changes and imbalances in the nigrostriatal dopaminergic release, along with functional and structural changes in the cortical-striatal network^48,49^. Such mechanisms may partially account for the diverse PD endophenotypes observed in our cohort, potentially influencing the development of motor (bradykinesia and gait impairment) and cognitive (visuospatial attention and verbal fluency) deficits. In support of this hypothesis our exploratory findings align with a growing body of evidence suggesting microstructural damage in the frontal cortex involving Brodmann area 6 and 8 (premotor cortex, supplementary motor area, the presupplementary motor area) in the early stages of PD^50,51^.

Importantly, superficial white matter represents a very vulnerable area, located beneath the infragranular layer of the cerebral cortex, containing the last fibers to myelinate and extensive cortico-cortical connections^29^ and has been already associated with ischemic and hemorrhagic damage during Covid-19 infection, cytotoxic lesions and deposition of Aẞ plaques, and may trigger epileptic seizures in patients with small vessel disease^25,52–53^.

Remarkably, the enrichment for superficial white matter hyperintensities was associated in *LRRK2* p.G2019S and *LRRK2* p.R1441C carriers with a trend toward an earlier age at onset, which could point to a potential increase of the penetrance of these variants.

Notably, *LRRK2* p.G2019S is a founder mutation with a high but incomplete penetrance, ranging from 15% to 80% and is most prevalent in Ashkenazi Jews and North African Berbers. Phenoconversion mechanisms have not been fully established however, *LRRK2* p.G2019S penetrance increases with age and is influenced by polygenic risk score^54^.

By contrast, and supporting our hypothesis, regular intake of aspirin, which is also prescribed for primary and secondary prevention of ischemic stroke, may be associated with reduced penetrance of *LRRK2*-associated PD variants^55^.

Analogously, Alzheimer’s disease patients carrying the nearly fully penetrant *PSEN1* mutations presented increased white matter hyperintensities at least 6 years before symptoms onset, with a burden which increases with greater temporal proximity to the estimated year of onset of clinical symptoms, likely promoting the penetrance of these variants^56^.

Additionally, approximately 30% of familial PD and prodromal PD patients displayed a bilateral enlargement of subcortical periventricular spaces particularly affecting the nucleus lentiformis and to a different extent the centrum semiovale and the thalamus and potentially indicating underlying glymphatic impairment (Fig. 4A) and appeared phenotypically linked to a mild rigidity of the neck and upper extremities while leaving cognitive functions largely unaffected. In line with these observations, previous studies have suggested a potential association between the severity of the rigidity-dominant subtype of motor symptoms in patients with PD and enlarged perivascular spaces, while similarly noting no significant difference in terms of cognitive performance^43^.

The glymphatic system has been implicated in alpha-synuclein clearance. In vivo experiments showed that in mice overexpressing the human *SNCA* p.A53T, AQP4-mediated glymphatic system impairment promoted the accumulation of alpha-synuclein, triggered the further loss of dopaminergic neurons and exacerbated parkinsonian-symptoms^57^, thus implying that the glymphatic activity may be a robust potential therapeutic target to delay PD progression^58^.

In addition, we observed an enrichment for lacunar infarcts in the basal ganglia and thalamus in the elderly familial PD patients. This finding appeared to parallel a mild increase of the MDS-UPDRS-III scores and hypokinetic-rigid syndrome. Importantly, lacunes have been reported to frequently occur in patients with PD, with a prevalence of approximately 50% ^59^ and were associated, in concert with our results, with mild parkinsonian signs and incident parkinsonism^59^.

We next tested the hypothesis that the main PD genetic causative and risk factors may explain the enrichment for cSVD neuroradiological biomarkers such as superficial white matter hyperintensities and lacunar infarcts in familial PD and prodromal patients, screening protein coding variability in *VPS35*, *DJ1*, *PINK1*, *ATP13A2*, *PRKN*, *SNCA*, *LRRK2*, *GBA*, *MAPT*, *LAMP3*, *STK39* in a cohort of 96 familial small vessel disease patients. We did not detect any pathogenic variant in the studied genes in this cohort. Notably, although the *LRRK2* p.G2019S variant has been reported to influence arterial cerebral blood flow in mouse models^6^, it was not observed in our cSVD cohort. Overall, arguing for a non-critical role of the main PD genes for the development and progression of cSVD within the sample studied.

Although these are exploratory observations that warrant confirmation in larger, longitudinal studies, our analysis supports a growing body of evidence pointing to vascular pathology as a potential PD modifier. Such cerebrovascular factors may likely influence both the onset and clinical spectrum, suggesting they may be plausible stratification factors when testing responses to potential therapeutic drugs. By integrating PPMI neuroimaging and genetic data in this familial PD cohort our study offers initial insights into possible cSVD-PD interactions and highlights a potential translational pathway for early intervention in PD. However, several methodological limitations deserve consideration : (1) the small sample size consisting of 104 familial PD-PD prodromal cases may restrict the generalizability of our observations; (2) the longitudinal MRI and comprehensive cardiovascular risk factor data were not uniformly available for all participants, limiting our ability to evaluate temporal progression; (3) the PPMI genetic analysis did not encompass protein coding variants in other PD Mendelian genes such as *PRKN*, *DJ1* and *PINK1* (autosomal recessive PD) and *VPS35* (autosomal dominant PD) nor did it include exonic rearrangements analysis; (4) cSVD and adult-onset leukodystrophy Mendelian genes were not specifically evaluated within this familial PD-PD prodromal cohort.

Early detection of cardiovascular phenotype in PD may eventually offer a complementary strategy to potentially delay PD symptom onset and mitigate associated motor and cognitive deficits. Consequently, these exploratory findings require further evaluation within larger longitudinal cohorts of familial PD and cSVD patients.

## Supplementary Information

Below is the link to the electronic supplementary material.

Supplementary Material 1.

Supplementary Material 2.

## Data Availability

All data generated or analysed during this study are included in this published article (and its Supplementary Information files). The PPMI datasets analysed during the current study is publicly available (www.ppmi-info.org)The genetic variants detected in the cSVD cohort have been submitted to Clinvar database (https://www.ncbi.nlm.nih.gov/clinvar/search/?assembly=GRCh38&terms=SUB16281935).
