# Supplementary material for: Exploring cerebral small vessel disease signatures in familial Parkinson’s disease
Source: Sci Rep. 2026 Sep 8;16:28063. doi: 10.1038/s41598-026-69989-z (PMC13550550; doi:10.1038/s41598-026-69989-z)
Supplement: Supplementary file 1 [file 41598_2026_69989_MOESM2_ESM.docx]

| **Feature** | **Vascular Parkinsonism** | | **Familial Parkinson’s disease** | | | **References** |
| --- | --- | --- | --- | --- | --- | --- |
|  | **Chronic familial cSVD (22%)** | **Acute sporadic subacute cSVD (2%)** | **AR** | **AD** | **Risk Factor** |  |
| **Genetics** | *COL4A1, FOXC1,* [*GLA*](https://panelapp.genomicsengland.co.uk/panels/50/gene/GLA/) *,* [*HTRA1,*](https://panelapp.genomicsengland.co.uk/panels/50/gene/HTRA1/) [*NOTCH3,* [*TREX1*](https://panelapp.genomicsengland.co.uk/panels/50/gene/TREX1/) *,*](https://panelapp.genomicsengland.co.uk/panels/50/gene/NOTCH3/) [*CST3*](https://panelapp.genomicsengland.co.uk/panels/50/gene/CST3/) *, SCN1A, SLC20A2* mutation  *EIF2B-*related disorder | Hypertension | *DJ1* and *PARK2* | *SNCA* | *LRRK2* G2019S | https://www.omim.org/ |
| **Age at onset** | 4–10 years later compared to patients with idiopathic Parkinson disease |  | Very early-onset, mean age at onset was 27 years | Early-onset, mean age at onset 46.5 years, and rapid progression | Late-onset, mean age at onset 65 years | https://www.omim.org/ |
| **Clinic** | **Cognitive**  dementia, cognitive impairment  **Motor: (Bilateral)**  -rapid course,  -akinesia, rigidity,  -rarely bradykinesia of the upper limbs, almost absence of the resting tremor, postural tremor,  -postural instability,falls  -pyramidal signs, pseudobulbar palsy, incontinence | **Cognitive:**  cognitive decline  **Motor**  Hemiparkinsonism/Parkinsonism | **Cognitive:**  cognitive decline  **Motor:**  juvenile Parkinson disease  retropulsion, dystonia of the feet, classic parkinsonism. Mild tremor, rigidity, and bradykinesia | **Cognitive:**  psychiatric features, cognitive decline  **Motor:**  bradykinesia and rigidity, central hypoventilation, postural hypotension, bladder incontinence, myoclonus, pyramidal signs | **Cognitive:**  No cognitive decline was noted in any of the patients even after long disease duration  **Motor:**  bradykinesia, rigidity, resting tremor, postural instability, and favorable response to levodopa.  decreased olfactory function and hyposmia | https://www.omim.org/ |
| **Neuroimaging (MRI/CT)** | MRI/CT: periventricular and subcortical white matter lesions (75–90%)  multiple territory infarcts (90-95%), basal ganglia ischemic lesions (38–44%).  -DAT-SPECT and IBZM-SPECT activity normal or little reduced | - Infarcts affecting left caudate, putamen and internal capsule  - Lacunar infarct affecting the substantia nigra | MRI:atrophy of the cerebellar hemisphere and vermis, as well as high intensity areas in both pyramidal tracts.  PET-Scan:  significantly decreased fluorodopa uptake in the caudate, putamen, and ventral and dorsal midbrain | normal brain MRI  SPECT scan showed decreased blood flow | PET-Scan:  progressive dopaminergic dysfunction affecting transport and uptake | https://www.omim.org/ |
| **Neuropathology** | Patchy areas of neuronal degeneration and loss, with free-lying nigral pigment and mild gliosis in the substantia nigra. Around the dilated perivascular space of the paramedian perforating branches, free-lying pigment and moderate proliferation of astroglial cells. Perivascular pallor, gliosis, hyaline thickening, and enlargement of perivascular spaces  Status cribrosus of the lateral striatal arteries in the putamina and substantia nigra bilateral |  | depigmentation of the substantia nigra, with severe loss of pigmented neurons in the pars compacta, deposition of extraneuronal melanin, and mild gliosis. No Lewy bodies or neurofibrillary tangles | depigmentation of the substantia nigra, severe cell loss and gliosis in the brainstem, and multiple alpha-synuclein-immunopositive Lewy neurites. Cortical neuritic changes associated with tissue vacuolization were present, mostly in the medial temporal regions. | pure nigral degeneration without Lewy bodies or neurofibrillary tangles | https://www.omim.org/ |

**Table S1. Main genetic, clinical, neuroimaging and neuropathological features of vascular parkinsonism and familial Parkinson’s disease.** cSVD, cerebral small vessel disease; AR, autosomal recessive; AD, autosomal dominant

| **Gene** | **cSVD (OMIM)** | **Ihn** | **Main clinical features** | **Parkinsonism** | **CT-MRI** | **Reference** |
| --- | --- | --- | --- | --- | --- | --- |
| [***CACNA1A***](https://panelapp.genomicsengland.co.uk/panels/50/gene/CACNA1A/) | Migraine, familial hemiplegic, 1 ([141500](https://www.omim.org/entry/141500)) | AD |  | rigidity, bradykinesia and a resting tremor, progressive gait instability,hypomimia, finger tapping and hand diadochokinesia | normal substantia nigra, bilateral loss of substance in the basal ganglia | (Bruun et al., 2015) |
| [***FOXC1***](https://panelapp.genomicsengland.co.uk/panels/50/gene/FOXC1/) | Axenfeld-Rieger Syndrome ([602482](https://www.omim.org/entry/602482)) | AD |  | Parkinson |  | (Zhang et al., 2016) |
| [***GLA***](https://panelapp.genomicsengland.co.uk/panels/50/gene/GLA/) | Fabry disease ([301500](https://www.omim.org/entry/301500)) | XL | Transient ischemic attacks (TIAs), strokes [2] and brain white matter lesions (WML) [3] are common central nervous system (CNS) manifestations. In recent years there has been growing body of evidence supporting a link between the pathogenesis of PD and lysosomal dysfunction, namely in Gaucher disease (GD) and FD. | Atypical Parkinsonism with axial signs (gait and postural instability), mild symmetrical rigidity and pyramidal signs (mild right hemiparesis, generalized hyperreflexia and positive Babinski sign on both sides | T2 Hyperintensities in the basal ganglia and deep white matter regions; Leukoencephalopathy with multifocal ischemic lesions including the head of the right caudate nucleus  deficiency on 18F-DOPA PET | (Gago et al., 2020), (Orimo et al., 1994), (Buechner et al., 2006) |
| [***HTRA1***](https://panelapp.genomicsengland.co.uk/panels/50/gene/HTRA1/) | CARASIL syndrome (only SVD) ([600142](https://www.omim.org/entry/600142)) | AR  AD |  | Extrapyramidal signs, Gait disturbance, Rigidity | Diffuse white matter abnormalities, Subcortical focal lacunae | https://www.omim.org/ |
| [***NOTCH3***](https://panelapp.genomicsengland.co.uk/panels/50/gene/NOTCH3/) | Cadasil (only SVD) ([125310](https://www.omim.org/entry/125310))  *-* p.R1006C  *-* p.C1315Y | AD | migraine, recurrent transient ischemic attacks or stroke, cognitive decline, psychiatric manifestations. Less frequent  manifestations of the disease are epilepsy, transient disturbances  of consciousness, visual impairment, and hemorrhagic  strokes and parkinsonism  moderate cognitive impairment (MMSE = 17/30) | slowly progressive parkinsonism, not responsive to l-dopa: akinesia, rigidity, and postural instability, symmetry of parkinsonism, cognitive impairment from moderate to severe, apraxia, pseudobulbar signs, urinary incontinence | T2-weighted images or FLAIR showed widespread ischemic lesions in the periventricular white matter, the internal and external capsules, the basal ganglia, and thalami. Multiple  subcortical lacunar infarcts and leukoencephalopathy extended to the external capsule on cerebral MRI suggested the presence of CADASIL. | (Ragno et al., 2013)^,^ (Valenti et al., 2011)^,^ (Chabriat et al., 2009)^,^ (Singhal et al., 2004)^,^ (Rufa et al., 2004)^,^ |
| [***TREX1***](https://panelapp.genomicsengland.co.uk/panels/50/gene/TREX1/) | Cadasil (only SVD) ([192315](https://www.omim.org/entry/192315)) | AD |  | extrapyramidal signs |  | https://www.omim.org/ |
| [***CST3***](https://panelapp.genomicsengland.co.uk/panels/50/gene/CST3/) | Cerebral amyloid angiopathy ([105150](https://www.omim.org/entry/105150)) | AD |  | Associated to MSA type |  | (Urbizu et al., 2015) |
| ***SCN1A*** | Migraine, familial hemiplegic, 3 ([609634](https://www.omim.org/entry/609634)) | AD |  | masked face and bradykinesia, resting tremor, hypertonia, antecollis, crouch gait, and bradykinesia, variable response to L-Dopa |  | (Kanatani et al., 2020) |
| ***SLC20A2*** | Basal ganglia calcification, idiopathic, 1 ([213600](https://www.omim.org/entry/213600)) | AD |  | severe bradykinesia, postural instability, mild symmetric rigidity without tremor, levodopa response | **CT** calcifications in the bilateral  basal ganglia, thalami, and dentate nuclei. Dopamine transporter (DAT) single photon emission CT shows diffusely decreased DAT density in the bilateral striatum. | (Ichikawa et al., 2019) |
| ***PRNP*** | Cerebral amyloid angiopathy, PRNP-related ([137440](https://www.omim.org/entry/137440)) | AD |  | Parkinsonism |  | (J et al., 2016), https://www.omim.org/entry/137440 |

**Table S2.** Main mendelian cerebral small vessel diseases leading to vascular parkinsonism. AD, autosomal dominant; AR, autosomal recessive; MSA, multisystem Atrophy

|  | **Periventricular Hyperintensities (PVH 0-6)** | **Scores** | **Significance of the scores** | **References** |
| --- | --- | --- | --- | --- |
|  | Capsule occipital  Capsule frontal  Bands lateral ventricles | 0/1/2  0/1/2  0/1/2 | 0 = absent  1 = < 15mm  2= > 15 mm | (Fazekas et al., 1987) |
|  | **Lobar Superficial White matter Hyperintensities (WMH 0-16)** |  |  |  |
|  | Frontal  Parietal  Occipital  Temporal | 0/1/2/3/4  0/1/2/3/4  0/1/2/3/4  0/1/2/3/4 | 1 = < 15mm, n ≤ 5  2= < 15 mm, n > 6  3 = > 15 mm, n ≤ 5  4 = > 15 mm, n > 6 | (Wang et al., 2022) |
|  | **Lobar Deep White matter Hyperintensities (WMH 0-20)** |  |  |  |
|  | Frontal  Parietal  Occipital  Temporal | 0/1/2/3/4/5  0/1/2/3/4/5  0/1/2/3/4/5  0/1/2/3/4/5 | 1 = < 15mm, n ≤ 5  2= < 15 mm, n > 6  3 = > 15 mm, n ≤ 5  4 = > 15 mm, n > 6  5 = confluent | (Fazekas et al., 1987) |
|  | **Deep White matter Hyperintensities (WMH 0-3)** |  |  |  |
|  |  | 0  1  2  3 | 0 = absent  1 = punctuate foci  2 = beginning confluence  3 = severe (large confluent areas) | (Fazekas et al., 1987) |
|  | **Status cribrosus (SC 0-2)** |  |  |  |
|  |  | 0  1  2 | 0 = Absent  1 = Mild/moderate  2 = Severe | (Ferrer et al., 1990)  (de Reuck et al., 1980) |
|  | **Lobar cortical small-microinfarcts (CMI 0-16)** |  |  |  |
|  | Frontal  Parietal  Occipital  Temporal | 0/1/2/3/4  0/1/2/3/4  0/1/2/3/4  0/1/2/3/4 | 1 = < 15mm, n ≤ 5  2= < 15 mm, n > 6  3 = > 15 mm, n ≤ 5  4 = > 15 mm, n > 6 | (Xiong et al., 2018) |
|  | **Lacunar Infarcts (LI. 0-1)** |  |  |  |
|  |  | 0  1 | 0 = Absent  1 = Present | (Wardlaw et al., 2019) |

**Table S3. Modified Scheltens scale used to analyse cerebral small vessel neuroradiological biomarkers in our cohort.** PVH, peri-ventricular hyperintensities; WMH, white matter hyperintensities; SC, status cribrosus; CMI, cortical microinfarcts; LI, lacunar infarcts

**MRI acquisition protocols**

**Axial T2 Flair MRI, 5mm, (average of 30 total slices), 100/153 PD-PD-prodromal cases and controls (65%)**

Acquisition Plane=AXIAL; Acquisition Type=2D; Coil=; Field Strength=3.0 tesla; Flip Angle=90.0 degree; Manufacturer=GE MEDICAL SYSTEMS; Matrix X=512.0 pixels; Matrix Y=512.0 pixels; Matrix Z=28.0 ; Mfg Model=Signa HDxt; Pixel Spacing X=0.5 mm; Pixel Spacing Y=0.5 mm; Pulse Sequence=SE/IR; Slice Thickness=5.0 mm; TE=120.9 ms; TI=2250.0 ms; TR=9502.0 ms; Weighting=T2.

**Axial FLAIR_LongTR T2, 4mm, (average of 33 total slices), 32/153 PD-PD-prodromal cases and controls (21%)**

Acquisition Plane=AXIAL; Acquisition Type=2D; Coil=B; Field Strength=3.0 tesla; Flip Angle=90.0 degree; Manufacturer=Philips Medical Systems; Matrix X=560.0 pixels; Matrix Y=560.0 pixels; Matrix Z=28.0 ; Mfg Model=Achieva; Pixel Spacing X=0.4 mm; Pixel Spacing Y=0.4 mm; Pulse Sequence=IR; Slice Thickness=4.0 mm; TE=125.0 ms; TI=2800.0 ms; TR=11000.0 ms; Weighting=T2.

**Axial T2 FLAIR, 3mm, (average of 49 total slices), 20/153 PD-PD-prodromal cases and controls (13%)**

Acquisition Plane=AXIAL; Acquisition Type=2D; Coil=; Field Strength=1.5 tesla; Flip Angle=90.0 degree; Manufacturer=GE MEDICAL SYSTEMS; Matrix X=512.0 pixels; Matrix Y=512.0 pixels; Matrix Z=56.0 ; Mfg Model=Signa HDxt; Pixel Spacing X=0.5 mm; Pixel Spacing Y=0.5 mm; Pulse Sequence=SE/IR; Slice Thickness=3.0 mm; TE=121.3 ms; TI=2000.0 ms; TR=8002.0 ms; Weighting=T2.

**Supplementary legends**

**Table S4.** Cohort description: familial PD, familial PD prodromal patients and controls. WMHCS, white matter hyperintensity cumulative score; LSWMHCS, lobar superficial white matter hyperintensity cumulative score, WMHCS, white matter hyperintensity cumulative score.

**Table S5.** White matter hyperintensity single and cumulative scores in familial PD, familial PD prodromal patients and controls.

**Table S6.** Longitudinal MRI, Moca, UPDRS III follow-up data for 14 PD and PD-prodromal patients

**Table S7.** Genetic screening of the main PD mendelian genetic causative and risk factors in 243 elderly controls from the HEX database.

**Figure S1**. Longitudinal MRI-MoCA-UPDRS III follow-up data available only for a minority of PD- and PD prodromal patients: 14 PD-PD Prodromal patients (13%, 1 PD and 13 PD-Prodromal patients). Average years between MRI scans are 4 (range 3-5). A mild increase of WMHCS (average of 3 [range 0-15] and particularly in the PD patient) corresponded to a proportional increase in UPDRS III (average 4 [range 0-14] and particularly in the PD patient). By contrast, MoCa scores remained mostly stable between MRI scans (average MoCA score difference of 0.4 [range 0-6]).PD, Parkinson’s disease; Pro, PD-Prodromal cases.

**Figure S2**. Relative frequency of rare protein coding variants in the most common PD causative genes (*VPS35*, *DJ1*, *PINK1*, *ATP13A2*, *PRKN*, *SNCA*, *LRRK2*) or genetic risk factors (*LRRK2, GBA*, *MAPT*, *LAMP3*, *STK39*) detected in 96 familial early-onset cerebral small vessel disease patients and 243 elderly neuropathologically proven controls (<https://www.alzforum.org/exomes/hex>). CSVD, cerebral small vessel disease; CTRLS, controls.

References

Bruun, M., Hjermind, L.E., Thomsen, C., Danielsen, E., Thomsen, L.L., Pinborg, L.H., Khabbazbavani, N., Nielsen, J.E., 2015. Familial hemiplegic migraine type 1 associated with parkinsonism: a case report. Case Rep Neurol 7, 84–89. https://doi.org/10.1159/000381827

Buechner, S., De Cristofaro, M.T.R., Ramat, S., Borsini, W., 2006. Parkinsonism and Anderson Fabry’s disease: a case report. Mov Disord 21, 103–107. https://doi.org/10.1002/mds.20675

Chabriat, H., Joutel, A., Dichgans, M., Tournier-Lasserve, E., Bousser, M.-G., 2009. Cadasil. Lancet Neurol 8, 643–653. https://doi.org/10.1016/S1474-4422(09)70127-9

Gago, M.F., Azevedo, O., Guimarães, A., Teresa Vide, A., Lamas, N.J., Oliveira, T.G., Gaspar, P., Bicho, E., Miltenberger-Miltenyi, G., Ferreira, J., Sousa, N., 2020. Parkinson’s Disease and Fabry Disease: Clinical, Biochemical and Neuroimaging Analysis of Three Pedigrees. J Parkinsons Dis 10, 141–152. https://doi.org/10.3233/JPD-191704

Ichikawa, Y., Tanaka, M., Kurita, E., Nakajima, M., Tanaka, M., Oishi, C., Goto, J., Tsuji, S., Chiba, A., 2019. Novel SLC20A2 variant in a Japanese patient with idiopathic basal ganglia calcification-1 (IBGC1) associated with dopa-responsive parkinsonism. Hum Genome Var 6, 44. https://doi.org/10.1038/s41439-019-0073-7

J, N., M, S., S, K., M, P., 2016. A Corticobasal Syndrome Variant of Familial Creutzfeldt-Jakob Disease with Stroke-Like Onset [WWW Document]. Case reports in neurological medicine. https://doi.org/10.1155/2016/4167391

Kanatani, M., Adachi, T., Sakata, R., Nishimura, Y., Saito, Y., Maegaki, Y., Watanabe, Y., Hanajima, R., 2020. Dravet syndrome with parkinsonian symptoms and intact dopaminergic neurons: A case report. Brain Dev. https://doi.org/10.1016/j.braindev.2020.10.015

Orimo, S., Iwasaki, T., Yoshino, H., Arai, M., Hiyamuta, E., 1994. [An autopsied case of Fabry’s disease presenting with parkinsonism and cardiomegaly as a cardinal clinical manifestation]. Rinsho Shinkeigaku 34, 1003–1007.

Ragno, M., Berbellini, A., Cacchiò, G., Manca, A., Di Marzio, F., Pianese, L., De Rosa, A., Silvestri, S., Scarcella, M., De Michele, G., 2013. Parkinsonism is a late, not rare, feature of CADASIL: a study on Italian patients carrying the R1006C mutation. Stroke 44, 1147–1149. https://doi.org/10.1161/STROKEAHA.111.000458

Rufa, A., De Stefano, N., Dotti, M.T., Bianchi, S., Sicurelli, F., Stromillo, M.L., D’Aniello, B., Federico, A., 2004. Acute Unilateral Visual Loss as the First Symptom of Cerebral Autosomal Dominant Arteriopathy With Subcortical Infarcts and Leukoencephalopathy. Archives of Neurology 61, 577–580. https://doi.org/10.1001/archneur.61.4.577

Singhal, S., Bevan, S., Barrick, T., Rich, P., Markus, H.S., 2004. The influence of genetic and cardiovascular risk factors on the CADASIL phenotype. Brain 127, 2031–2038. https://doi.org/10.1093/brain/awh223

Urbizu, A., Canet-Pons, J., Munoz-Marmol, A.M., Aldecoa, I., Lopez, M.T., Compta, Y., Alvarez, R., Ispierto, L., Tolosa, E., Ariza, A., Beyer, K., 2015. Cystatin C is differentially involved in multiple system atrophy phenotypes. Neuropathol Appl Neurobiol 41, 507–519. https://doi.org/10.1111/nan.12134

Valenti, R., Bianchi, S., Pescini, F., D’Eramo, C., Inzitari, D., Dotti, M.T., Pantoni, L., 2011. First report of a pathogenic mutation on exon 24 of the NOTCH3 gene in a CADASIL family. J Neurol 258, 1632–1636. https://doi.org/10.1007/s00415-011-5983-3

Zhang, L., Deng, J., Pan, Q., Zhan, Y., Fan, J.-B., Zhang, K., Zhang, Z., 2016. Targeted methylation sequencing reveals dysregulated Wnt signaling in Parkinson disease. J Genet Genomics 43, 587–592. https://doi.org/10.1016/j.jgg.2016.05.002
